# Single Dose of Consensus Hemagglutinin-Based Virus-Like Particles Vaccine Protects Chickens against Divergent H5 Subtype Influenza Viruses

**DOI:** 10.3389/fimmu.2017.01649

**Published:** 2017-11-27

**Authors:** Peipei Wu, Jihu Lu, Xuehua Zhang, Mei Mei, Lei Feng, Daxin Peng, Jibo Hou, Sang-Moo Kang, Xiufan Liu, Yinghua Tang

**Affiliations:** ^1^Institute of Veterinary Immunology & Engineering, Jiangsu Academy of Agricultural Sciences, Nanjing, China; ^2^National Research Center of Engineering and Technology for Veterinary Biologicals, Jiangsu Academy of Agricultural Sciences, Nanjing, China; ^3^Jiangsu Co-Innovation Center for the Prevention and Control of Important Animal Infectious Disease and Zoonosis, Yangzhou, China; ^4^Animal Infectious Disease Laboratory, College of Veterinary Medicine, Yangzhou University, Yangzhou, China; ^5^Center for Inflammation, Immunity & Infection, Institute for Biomedical Sciences, Georgia State University, Atlanta, GA, United States

**Keywords:** H5 subtype influenza virus, vaccine, consensus, virus-like particles, broad protection

## Abstract

The H5 subtype highly pathogenic avian influenza (HPAI) virus is one of the greatest threats to global poultry industry. To develop broadly protective H5 subunit vaccine, a recombinant consensus HA sequence (rHA) was constructed and expressed in virus-like particles (rHA VLPs) in the baculovirus-insect cell system. The efficacy of the rHA VLPs vaccine with or without immunopotentiator (CVCVA5) was assessed in chickens. Compared to the commercial Re6 or Re6-CVCVA5 vaccines, single dose immunization of chickens with rHA VLPs or rHA-CVCVA5 vaccines induced higher levels of serum hemagglutinin inhibition titers and neutralization titers, mucosal antibodies, IFN-γ and IL-4 cytokines in sera, and cytotoxic T lymphocyte responses. The rHA VLPs vaccine was superior to the commercial Re6 vaccine in conferring cross-protection against different clades of H5 subtype viruses. This study reports that H5 subtype consensus HA VLP single dose vaccination provides broad protection against HPAI virus in chickens.

## Introduction

The H5 subtype highly pathogenic avian influenza (HPAI) viruses not only affect millions of domestic poultry, including chickens, ducks, and geese, as well as thousands of migratory wild birds, but also risk to the public health. Since the H5 subtype HPAI viruses emerged in Southeast Asia in 1990s and have evolved into 10 phylogenetic clades (0–9) and more than 30 subclades based on their hemagglutinin (HA) genes (1, 2). Recently, H5 HPAI viruses of subclade 2.3.4 revealed a novel propensity to reassort with neuraminidase (NA) subtypes other than N1, including H5N2, H5N5, H5N6, and H5N8 (3–5).

A strategy of culling in combination with vaccination is a major current measure to prevent and control the spread of H5 HPAI viruses in several countries and regions (6–8). In China, a series of recombinant commercial vaccines, Re1, Re3–Re8, Re10, D7, and D8, have been developed and updated since 2004 to ensure an antigenic match between the vaccines and the circulating strains (8). However, H5 subtype HPAI viruses are still the pathogen with leading potential threat for poultry industry in China. It has been an everlasting difficult situation that the existing vaccine does not provide optimal protection against the emergence of new variants. The rapid mutation of the H5 viruses continues to bypass the time-consuming procedures of screening vaccine candidates during development of traditional inactivated whole-virus vaccine. Besides, propagation of influenza virus in the embryonated chicken eggs during preparation of inactivated virus vaccine produces biohazardous waste materials and leads potential threat to animal and human public health.

In this regard, there is an urgent need for developing an effective, broadly cross-protective, and safe H5 vaccine for use in poultry farms. Consensus sequences contain the most common residue at each position after aligning with a population of sequences. Previous studies reported that the consensus HA-based influenza vaccine candidates have elicited cross-reactive immune responses (9–12). There are commercial trivalent HA protein vaccines consisting of three insect-cell-derived full-length HAs similar to the seasonal trivalent inactivated vaccine, which represents an alternative to chicken-egg derived vaccines. In this study, we developed an insect-cell-derived consensus H5 subtype HA gene based virus-like particles (rHA VLPs) vaccine. Single dose immunization of chickens with rHA VLPs vaccines elicited serum and mucosal antibodies and cell-mediated immune responses at high levels, and induced cross-protection against subclade 2.3.4.6 and clade 7 of H5 HPAI viruses.

## Materials and Methods

### Cell Lines, Viruses

*Spodoptera frugiperda* Sf9 insect cells (CRL-1711; ATCC, Manassas, VA, USA) were maintained in serum-free SF900II medium (Invitrogen, Carlsbad, CA, USA) at 27°C and used for production of recombinant baculoviruses (rBVs) and VLPs. Two HPAI strains (ZJ and DT) used as challenge virus were originated from clade 2.3.4.6 (A/Chicken/Zhejiang/2011, ZJ, H5N2) and clade 7 (A/Chicken/Huadong/4/2008, DT, H5N1). In this study, the recombinant Re6 commercial vaccines, and chicken sera against monovalent Re5, Re6, Re7, and Re8 antigens were purchased from Weike Biotechnology Co., Ltd., Harbin, China. Four commercial recombinant vaccines of Re5, Re6, Re7, and Re8 were inactivated whole-virus-based mineral oil emulsion vaccines (Weike). The six inner genes of four recombinant vaccine strains are derived from A/Puerto Rico/8/1934 virus (PR8, H1N1). The HA and NA genes of Re5–Re8 are derived from different subclades of H5 wild-type viruses, Re5 from strain of A/duck/Anhui/1/2006 (clade 2.3.4) (8), Re6 from virus of A/duck/Guangdong/S1322/2006 (clade 2.3.2) (8), Re7 from strain of A/Chicken/Liaoning/S4092/2011 (clade 7.2) (13), and Re8 from A/chicken/Guizhou/4/13 (clade 2.3.4.4) (14).

### Preparation of Consensus HA Gene-Based Recombinant Proteins

It is known that nucleotide changes are more often because of non-sense mutations. Thus, the frequency of mutations at the nucleotide level is higher than that of the amino acid changes. Other researchers have also reported the consensus sequence derived from the nucleotide level (15). The complete open reading frame (ORF) of consensus HA was derived from the consensus nucleotide sequences of the H5 subtype HA after mega-aligning available complete H5 subtype HA sequences which isolated from 2004 to 2012 in China and originated from avian species in GenBank with Clustal V program (Lasergene, v.11; DNAStar, Madison, WI, USA). A total number of 304 HA sequences were analyzed in this study and the highest frequency of nucleotides in each position was selected to generate the consensus HA. The additional leucine zipper GCN-pII sequence was fused to the C-terminal end of the selected HA gene to form trimers as described (16). This engineered recombinant HA was named as rHA. In the results of the complete HA ORF consensus sequence after mega-aligning, the most adaption region is the HA1 head domain (61–161 aa), compared to the relatively conserved HA2 domain during evolution. The codon of the rHA ORF was optimized according to the codon usage frequency of *S. frugiperda* cell by Gene Script Co. Ltd (Nanjing, Jiangsu, China).

The optimized rHA ORF genes were cloned into the pFastBac vector plasmid to make recombinant Bacmid baculovirus DNAs using DH10Bac competent cells (rAcNPV, Invitrogen, Carlsbad, CA, USA). A recombinant baculoviruses (rBVs) expressing influenza rHA protein was generated by transfection of *Sf*9 insect cells according to the manufacturer’s instruction. *Sf*9 cells were infected (1.0 multiplicity of infection, MOI) with rBVs expressing HA. After 4 days, the cell culture supernatants were harvested for preparation of vaccines.

### Analysis of the Recombinant HA Proteins with Western Blot

The indirect immunofluorescence assay (IFA) was carried out to test the expression of HA in infected *Sf9* cells after 72 h. The primary antibody was chicken anti-sera against Re6 antigen at a dilution of 1:800, and the secondary antibody was FITC labeled goat anti-chicken IgY (1:500) (Southern Biotech, Birmingham, AL, USA).

The infected insect cells were harvested after inoculation of a single rHA rBVs for 96 h, and disrupted by ultrasonic for 30 min to prepare cell lysates under the condition of maintaining the temperature at 0–4°C. The sonicated cell lysates were cleared by low-speed centrifugation (10,000 × *g* for 20 min at 4°C) to remove cell debris. The rHA VLPs in the supernatants were pelleted by ultracentrifugation (100,000 × *g* for 60 min). The sedimented particles were resuspended in phosphate-buffered saline (PBS) at 4°C overnight and further purified through a 20–30–60% discontinuous sucrose gradient at 100,000 × *g* for 2 h at 4°C (17).

For Western blot analysis to determine the expression of rHA, infected cells were dissolved in sodium dodecyl sulfate (SDS)-polyacrylamide gel electrophoresis (PAGE) sample buffer (50 mM Tris, 3% β-mercaptoethanol, 2% SDS, 10% glycerol), separated by SDS-PAGE, and then transferred to nitrocellulose membranes, subsequently probed with sera (1: 1,000) derived from Re5 and Re6 vaccine-immunized chickens, respectively. The secondary antibodies were HPR-labeled goat anti-chicken IgY (1:2,000) (SoutherBiotech, Birmingham, AL, USA).

The functionality of rHA protein incorporated into VLPs was quantified by hemagglutination assay (HA assay) using 1% (v:v) chicken red blood cells. The concentration of protein was measured by Pierce BCA Protein Assay Kit (Thermo Fisher Scientific).

### Electron Microscopy

For negative staining of VLPs, sucrose gradient-purified VLPs were applied to a carbon-coated Formvar grid for 30 s. Excess VLPs suspension was removed by blotting with filter paper, and the grid was immediately stained with 1% phosphotungstic acid for 90 s. Excess stain was removed by filter paper, and the samples were examined using a transmission electron microscope.

### Vaccine and Immunopotentiators

The purified recombinant HA proteins were recovered to same volume before ultracentrifugation treatment. The diluent is phosphate buffer solution (pH 7.2, PBS). The rHA (ca. 12 µg/dose) were prepared as water-in-oil emulsion with or without immunopotentiator CVCVA5, named as rHA or rHA-CVCVA5, respectively. The rHA antigen stock solutions were normalized so that the vaccine preparations had the same reciprocal of hemagglutination titers equal to or higher than 256 as measured with 1% chicken red blood cells. This range of titers is a prerequisite for the monovalent Re6 vaccine preparations with mineral oil-in-water emulsion adjuvant in the manufacture company. The preparation of rHA-CVCVA5 was followed as described in our previous reports (18–20). Briefly, the components of CVCVA5 are composed of an aqueous phase that contains L–D isoform muramyl dipeptide (MDP) (InvivoGen), poly I:C (InvivoGen), levamisole hydrochloride (Sigma) added to rHA antigen solution as well as an oil phase of resiquimod (InvivoGen) and imiquimod (InvivoGen) added to Marcol 52 mineral oil (ESSO). One volume aqueous phase was mixed with three volumes of an oil phase. The rHA vaccine preparation in water-in oil emulsion did not contain the adjuvant ingredients of CVCVA5. Preparation of the emulsion form of CVCVA5 was similar to that of the rHA-CVCVA5, having PBS substituted with the rHA antigen solutions and 10-fold concentration of the ingredients of the CVCVA5 as those in the rHA-CVCVA5. The H5-CVCVA5 vaccines were prepared by mixing the commercial H5 (Re6, Weike) subtype vaccine (v/v, 1:9) with an emulsion form of CVCVA5.

### Immunization and Challenge

Groups (*n* = 25 in each group) of 3-week-old age specific pathogen-free (SPF) chickens (Leghorn breed, *Gallus gallus domesticus*) or 15- to 18-day-old age Hy Line Brown breed commercial chickens (*Gallus gallus domesticus*) were used to determine the immunogenicity and efficacy of the recombinant rHA VLPs. The vaccine test groups included the rHA with or without of CVCVA5. The commercial H5 subtype vaccines (Re6) were set as comparison control with or without CVCVA5, and a group of unvaccinated chickens as a naïve control. Serum samples from all birds were collected at 2 and 3 weeks postvaccination (PV).

Chickens from each test group were randomly selected and intranasal challenged with 100 LD_50_ (0.1 ml) H5 wild-type influenza viruses (ZJ and DT) at 3 weeks PV. These challenge viruses were derived from the clade of 2.3.4.6 (A/Chicken/Zhejiang/201, ZJ, H5N2, *n* = 10 in each challenge group) and clade 7 (A/Chicken/Huadong/4/2008, DT, H5N1, *n* = 10 in each challenge group) (19). Chickens were monitored and recorded clinical signs and body weights for 14 days post challenge (PC). All survival chickens were killed humanely at the end of monitoring experiments. Oropharyngeal and cloacal swab samples were collected at 3, 5, and 7 days PC, or collected from the dead chickens during the observation period. Virus isolation from the swab samples was performed as previously described (21).

### Antibody and Cytokine Tests

Serum antibody levels were titrated by hemagglutinin inhibition (HI) assay or serum-neutralization (SN) assay in DF-1 cell (ATCC, CRL-12203) (19, 22). The SN assay was performed as following described. Briefly, all serum samples from chickens were heat-inactivated (56°C, 30 min). The twofold serial dilutions of serum samples were mixed with equal volumes of 200 mean tissue culture infective dose (TCID_50_) of H5 subtype variant viruses (ZJ, DT). After 1 h incubation at 37°C, the mixtures were inoculated into the 96-well plates with a monolayer of DF-1 cells, a chicken embryo fibroblasts cell line, and then incubated to observe cytopathic effects (CPE) daily for up to 5 days. The cell death or CPE were used to determine the end-point titers that were calculated as the highest reciprocal dilution of sera at which virus infection is blocked in DF-1 cells according to the method of Reed and Muench (23).

The chicken cytokine levels of IFN-γ (Invitrogen, CA, USA) and IL-4 (USCN, Wuhan, China) in sera at 2 and 3 weeks PV were measured by commercially available ELISA kits following the manufacturer’s instructions as described in our previous reports (19, 20). The mucosal antibodies from bronchoalveolar lavage fluids (BAL) were determined by an HI assay.

### Cytotoxic T Lymphocytes (CTL) Assay

Cytotoxic T lymphocytes activity was measured by the lactate dehydrogenase (LDH) cytotoxicity assay kit (Takara, Kyoto, Japan), which is a non-radioactive method to detect the stable cytosolic enzyme LDH released from lysed cells. The assay was performed with the manufacturer’s instruction and our previous study (19, 20). Briefly, the target cells that derived from the inbred SPF chickens (B12/B12) embryo fibroblast cells were infected for 8 h with ZJ or DT virus with MOI of 0.1. The target cells (10^4^ cells in 100 µl/well) infected or uninfected were also seeded to each well. The effector cells derived from peripheral blood mononuclear cells isolated from the inbred SPF chickens at 21 days PV, which previously immunized with H5 rHA vaccine or the commercial H5 subtype vaccine (Re6) with or without CVCVA5 adjuvants, respectively. Various amounts of effector T cells were added to each well. Each cell sample was plated in triplicates. Microtiter plates were centrifuged at 250 × *g* for 5 min and incubated in a humidified chamber at 37°C, 5% CO_2_. After 4-h incubation, the supernatants were harvested, followed by the addition of the substrate tetrazolium salt. The OD values were read at 490 nm in an enzyme-linked immunosorbent assay reader. The specific LDH activity release was calculated by using the following formula: (experimental release − spontaneous release)/(maximum release − spontaneous release) × 100.

### Statistics

Experimental data are presented as mean ± SD of the mean. Prism 7 (GraphPad Software, Inc., San Diego, CA, USA) was used for data analysis. The statistical significance was analyzed with Student’s *t*-test or a one-way analysis of variance. Comparisons used to generate *P* values are indicated by horizontal lines (*P < 0.05; **P < 0.01; ***P < 0.001).

### Ethics Statement

All animal studies were carried out in accordance with the recommendations in the National Guide for the Care and Use of Laboratory Animals. The protocol (VMRI-AP150306) was approved by the Review Board of National Research Center of Engineering and Technology for Veterinary Biologicals, Jiangsu Academy of Agricultural Sciences and Yangzhou University. The surgery and euthanasia were performed under anesthesia with sodium pentobarbital solution (100 mg/kg body weight) *via* an intravenous route to minimize suffering. All experiments involved in the live H5 subtype viruses were performed in the biosafety level 3 laboratory facilities. At the end of experiments, the discarded live viruses, wastes, and infected animal carcasses were autoclaved and incinerated to eliminate biohazards.

## Results

### Production and Analysis of rHA-Based Influenza VLPs

**Figure 1:**
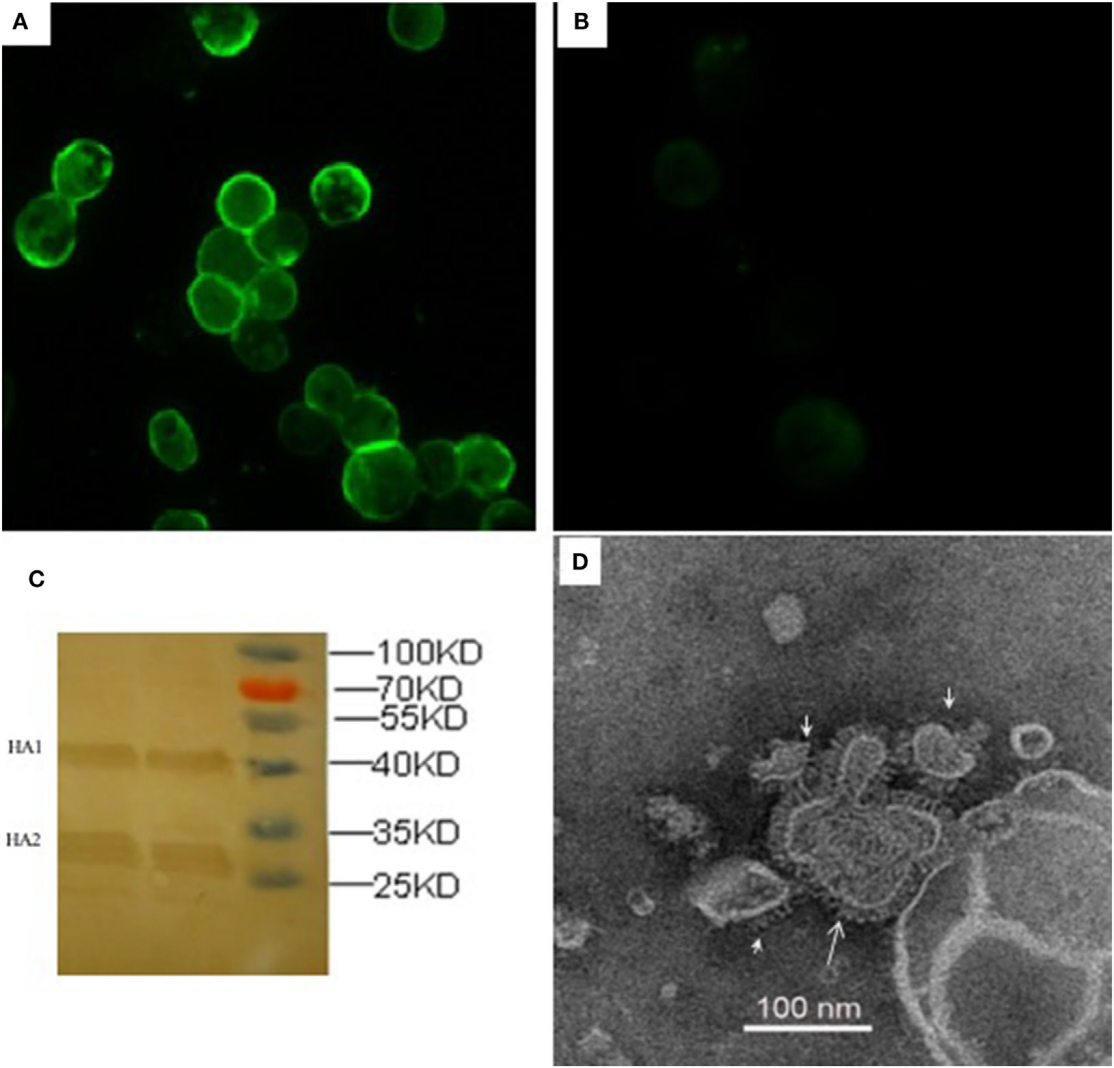
Characterization of recombinant consensus HA (rHA) proteins by indirect immunofluorescence assay (IFA), western blotting, and electron microscopy. IFA was analyzed in *Sf*9 cells infected with recombinant baculoviruses (rBVs) of HA **(A)** or only empty baculoviruses **(B)** after 72 h. The antibodies used in the test include chicken sera of anti-H5 (Re6 vaccine) for the primary reaction and goat anti-chicken IgY for the secondary antibody detection. **(C)** Western blotting analysis of the expression of recombinant HA proteins. **(D)** Negative staining electron microscopy of rHA-based influenza VLPs produced from the *Sf*9 cells infected with rBVs expressing a single consensus recombinant HA gene.

The recombinant HA proteins were produced in insect cells, which were infected with rBVs expressing the H5 subtype AIVs consensus HA gene with poly-basic residues at the cleavage site. The expression of rHA proteins was observed with indirect IFA in *Sf*9 insect cells 72 h after infection with rHA rBVs (Figure 1), whereas there was no specific fluorescence in control baculovirus infected cells (Figure 1). As for further evidence, two major visible bands, HA1 and HA2 fragments, were presented in the Western blot analysis which is consistent with a previous study (24). The functionality of rHA incorporated into VLPs (rHA VLPs) was assessed by the hemagglutination assay with 1% cRBC. No activity of hemagglutination was observed when the VLPs were denatured by heat treatment at 100°C for 10 min (data not shown). The reciprocal hemagglutinin titers of the rHA rBV-infected *Sf*9 cell culture supernatants reached to 64 before treating with ultrasonic, and increased to 2,048 after ultrasonic treatment. The increment in HA titers was speculated due to the release of rHA VLPs from the cellular membranes after the ultrasonic treatment of cell lysis. The total protein concentration of culture supernatants was 120 µg/mL measured by the BCA kit.

The size and morphology of rHA VLPs were examined by electron microscopy (Figure 1). The morphology of VLPs resembles that of influenza virus particles, and the spikes were observed on their surfaces which is characteristic of influenza virus HA proteins on virions.

### H5 rHA Displays Cross-Reactivity to Immune Sera Induced by H5 Vaccines

**Figure 2:**
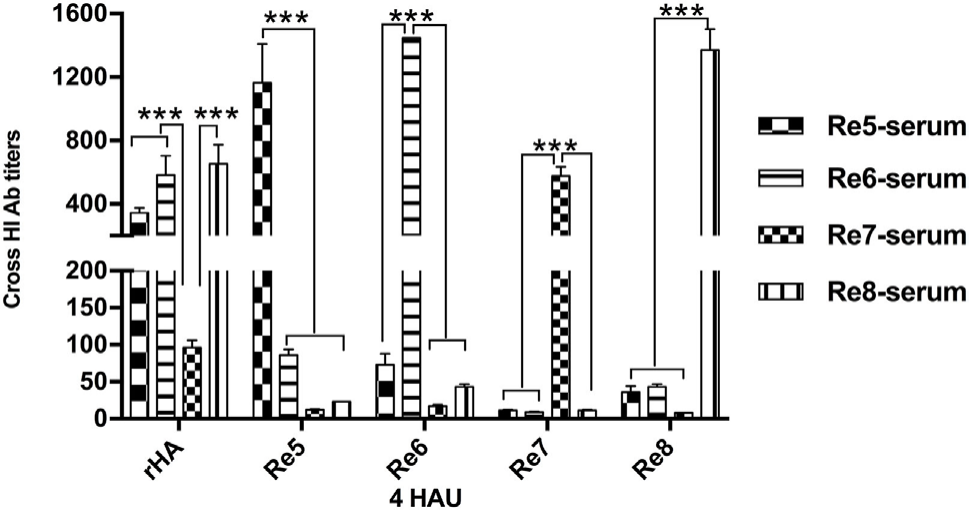
Hemagglutinin inhibition (HI) cross-reactivity of H5 rHA protein to sera raised by different vaccines. The HI tests were carried out with the recombinant protein of consensus H5 HA (rHA), viral antigens of Re5, Re6, Re7, and Re8 as 4 hemagglutinin units (HAU)/25 μl in three replicates. The six inner genes of Re5, Re6, Re7, and Re8 vaccine strains are derived from A/Puerto Rico/8/1934 virus (PR8, H1N1), the HA and neuraminidase genes of Re5 from strain of A/duck/Anhui/1/2006 (clade 2.3.4), of Re6 from virus of A/duck/Guangdong/S1322/2006 (clade 2.3.2), of Re7 from A/Chicken/Liaoning/S4092/2011 (clade 7.2), and of Re8 from A/chicken/Guizhou/4/13 (clade 2.3.4.4). The statistical significance was analyzed with Student’s *t*-test. ***P < 0.001.

The antigenicity of H5 rHA vaccine was determined *via* HI tests in cross reactions with sera raised by antigenically different H5 vaccines, and compared to those reactions with the viral antigens of Re5, Re6, Re7, and Re8. Except the HI titers from the homologous pairs of serum and antigen, the HI titers against the rHA antigen were higher than those that of the viral antigens, the Re5, Re6, Re7, and Re8, cross reacted with sera induced by Re5, Re6, Re7, and Re8 vaccines, respectively. Compared to the sera derived from Re5, Re6, and Re8 vaccines, the serum derived from Re7 vaccine was poorly reactive to rHA and other viral antigens (Figure 2).

### H5 rHA VLPs Vaccines Elicit Immune Responses in Chickens

**Figure 3:**
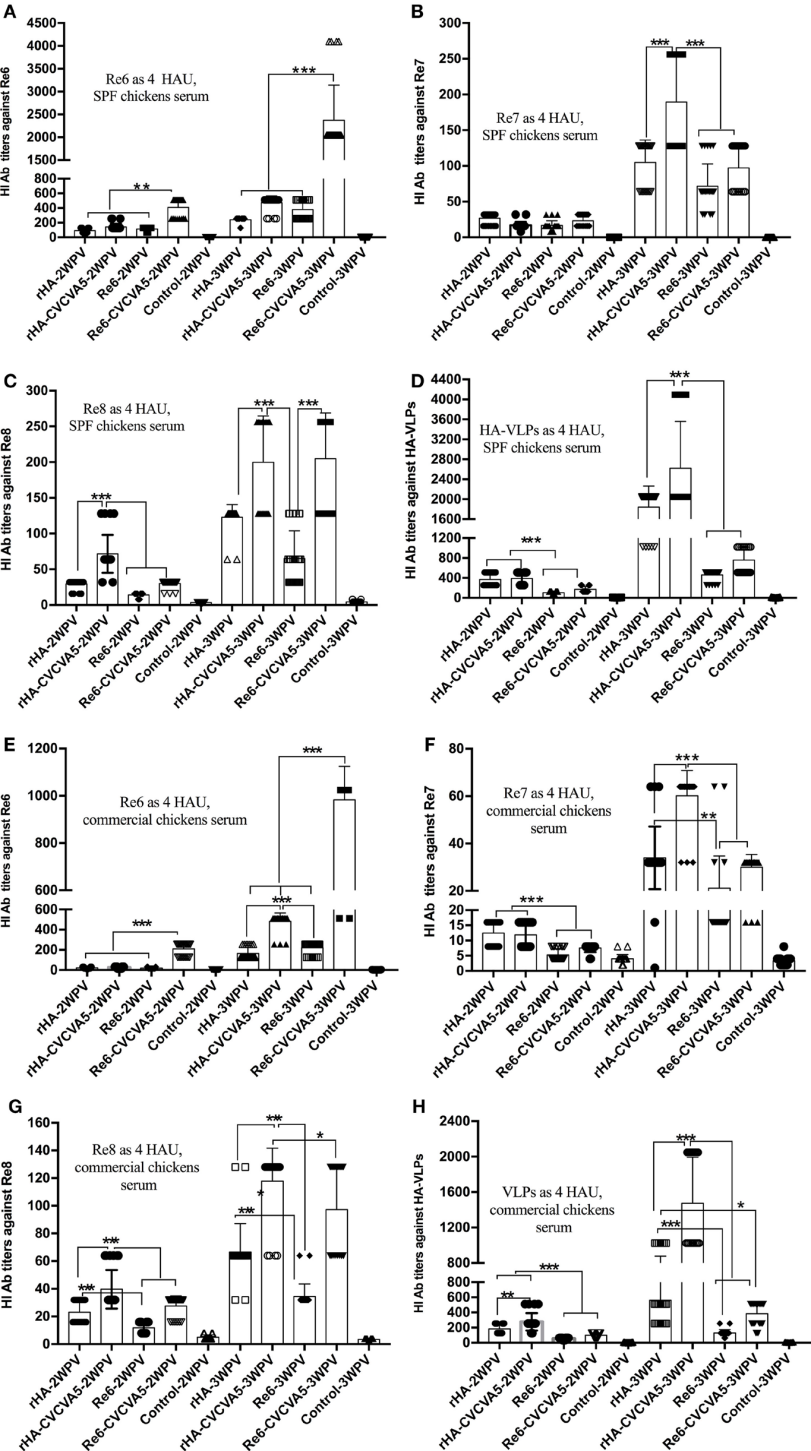
Hemagglutinin inhibition (HI) titers of immune sera from vaccinated specific pathogen-free (SPF) and commercial chickens. The SPF **(A–D)** or commercial **(E–H)** chickens were subcutaneously immunized with rHA VLPs vaccine with (rHA-CVCVA5) or without immunopotentiator (rHA), or Re6 vaccine with (Re6-CVCVA5) or without immunopotentiator (Re6). The serum samples were collected at 2- and 3-week postvaccination. The HI titers of SPF chickens sera were measured with 4 HAU testing antigens of Re6 **(A)**, Re7 **(B)**, Re8 **(C)**, and rHA **(D)**, and commercial chickens sera with 4 HAU antigens of Re6 **(E)**, Re7 **(F)**, Re8 **(G)**, and rHA **(H)**. The statistical significance was analyzed with Student’s *t*-test or a one-way analysis of variance. **P < 0.01, ***P < 0.001.

To determine whether influenza H5 rHA VLPs induce immune responses specific to influenza virus HA protein, groups of 3-week-old SPF chickens were subcutaneously vaccinated one time with influenza rHA vaccine (12 µg/dose) with or without immunopotentiator (rHA, rHA-CVCVA5), and Re6 vaccine in the presence or absence of immunopotentiator as controls (Re6, Re6-CVCVA5). The levels of serum antibody against different antigens were measured by HI assay at 2 and 3 weeks after a single dose vaccination. Using 4 HA units (HAU) of Re6 as a testing antigen, the HI titers of chickens shot with the Re6 vaccine were higher than those that induced by the rHA vaccine, similarly, HI titers induced by Re6-CVCVA5 vaccine were higher than those by the rHA-CVCVA5 vaccine at 2- or 3-week PV (Figure 3A). However, the HI titers raised by vaccination with rHA-containing vaccines were higher than those induced by Re6-containing vaccines when measured with the 4 HAU of Re7, Re8, or rHA testing antigens, respectively (Figures 3B–D).

The efficacy of rHA vaccines was also assessed in 15- to 18-day-old commercial chickens. Consistent with the results of SPF chickens, lower HI levels were observed in the rHA-containing vaccine groups compared to those in the Re6-containing vaccines groups, as measured by using 4 HAU of the Re6 as a testing antigen. In contrast, the HI antibody titers in rHA-containing vaccine groups were higher than those of the Re6-containing vaccine groups when determined with the rHA and Re7 antigens, respectively. Similar levels of HI antibodies against the Re8 antigen were observed in chickens which received Re6- or rHA-containing vaccines (Figures 3E–H).

### H5 rHA VLPs Vaccination Induces Enhanced Levels of Serum Virus Neutralization Titers and Mucosal Antibodies in SPF Chickens

**Figure 4:**
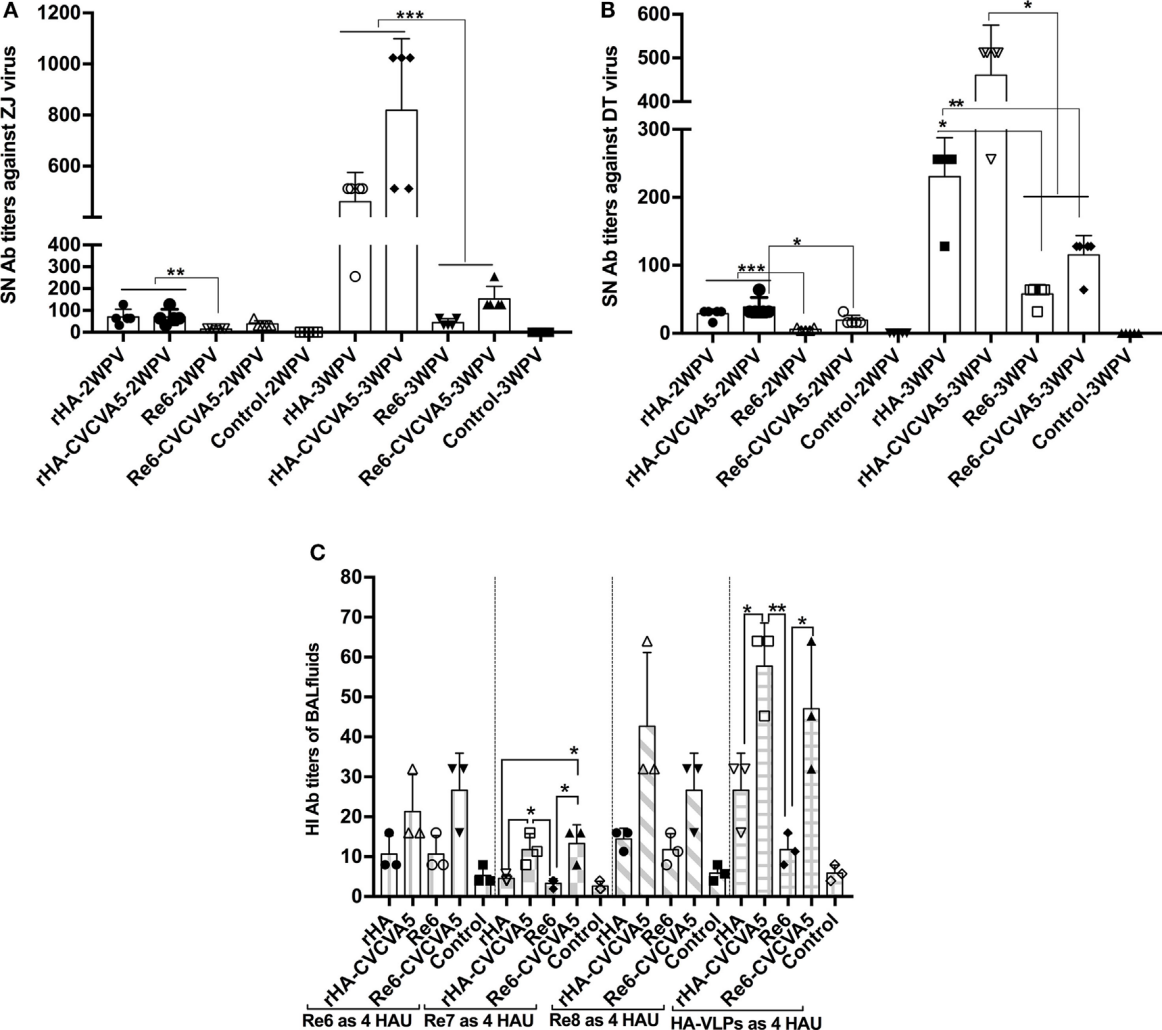
Serum-neutralization (SN) titers and hemagglutinin inhibition (HI) antibody titers in bronchoalveolar lavage (BAL) fluids. SN assays were performed in DF-1 cells. A fixed viral solution (200 TCID_50_, ZJ, or DT viruses) was reacted with the same volume of twofold serial diluted serum solutions. The pooled (five pooled in one) sera samples (*n* = 5) were derived from each group of the specific pathogen-free chickens vaccinated with rHA or Re6 vaccine with or without adjuvant. Both DT (clade 7) and ZJ (subclade 2.3.4.6) viruses were isolated from chickens **(A,B)**. The mucosal antibodies derived from BAL fluids (*n* = 3) were measured by HI assay with 4 HAU of Re6, Re7, Re8, and rHA at 3-week postvaccination **(C)**. The statistical significance was analyzed with Student’s *t*-test or a one-way analysis of variance. **P < 0.01, ***P < 0.001.

Serum-neutralization activity against the variants of ZJ or DT virus strains was carried out. Serum samples from the chickens that were immunized with rHA VLPs vaccine in the presence or absence of CVCVA5 and Re6 plus CVCVA5 vaccines showed significantly higher neutralization titers than those of the chickens that were immunized with Re6 vaccine without immnopotentiators at 2 and 3 weeks PV (Figures 4A,B).

The mucosal antibody titers in the BAL fluids at 3 weeks PV were also measured by HI assay with Re6, Re7, Re8, and rHA VLPs as 4 HAU antigens. Higher HI titers in BAL fluids from the rHA-CVCVA5 or Re6-CVCVA5 vaccine groups were observed compared to those groups that received rHA or Re6 vaccine without immunopotentiators (Figure 4C).

### H5 rHA VLPs Vaccination Induces Increased Levels of Serum Cytokines and CTL Responses in SPF Chickens

**Figure 5:**
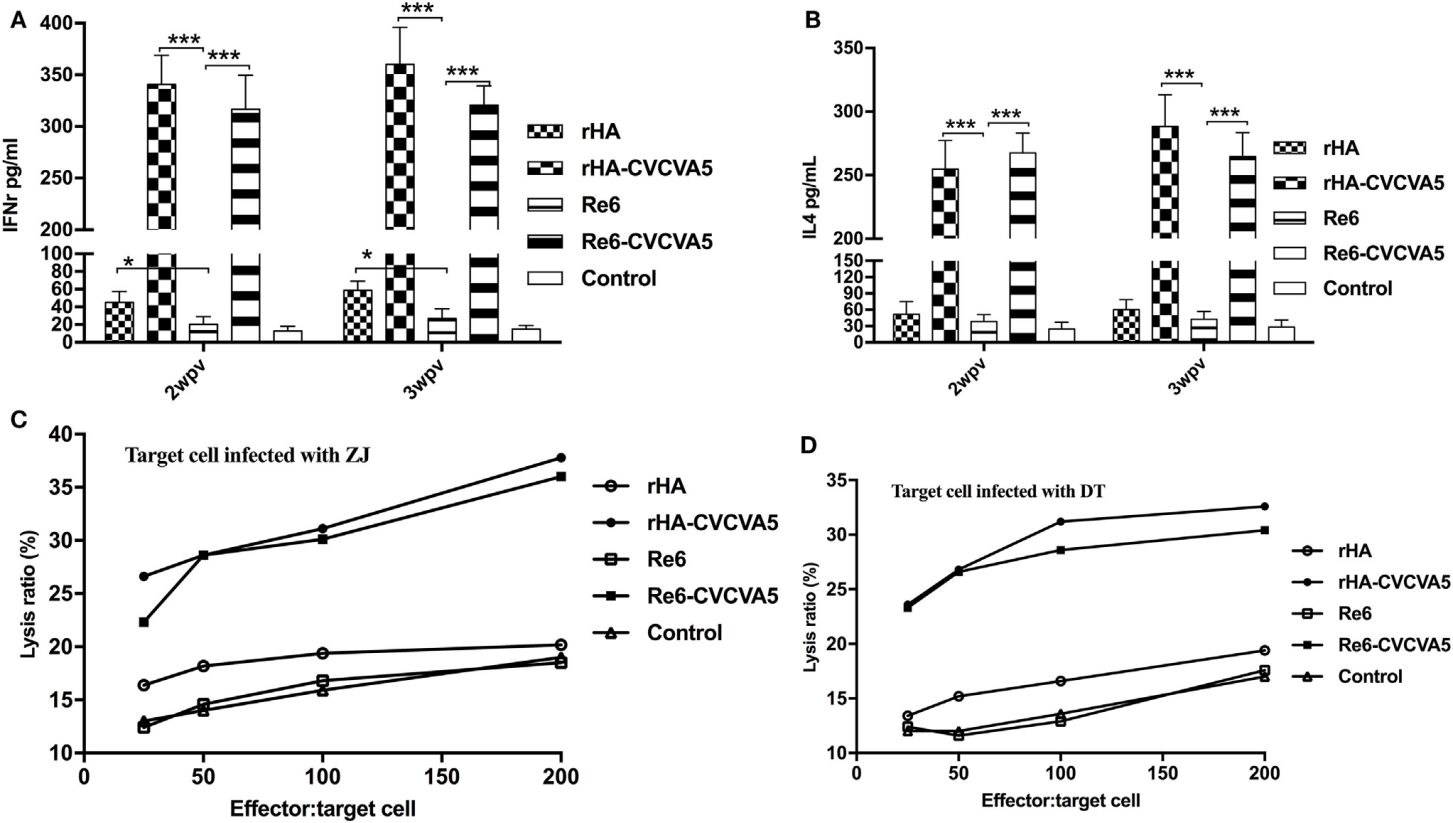
Levels of cytokines in sera and cytotoxic T lymphocytes activity in peripheral blood mononuclear cells after vaccination. The cytokines levels of IFN-γ **(A)** and IL-4 **(B)** in specific pathogen-free (SPF) chickens sera were determined by ELISA Kits at 2- and 3-week postvaccination from each group (*n* = 3). Cytotoxic T cells were derived from vaccinated inbred chickens. Inbred chicken embryo fibroblasts infected with DT or ZJ H5 subtype AI viruses were used as target cells. PBMCs derived from the SPF chickens vaccinated with rHA or Re6 vaccine with or without adjuvants, or from control group, were used as effector cells **(C,D)**. *P < 0.05, ***P < 0.001.

To investigate the induction of cytokines after vaccination, we measured the levels of IFN-γ and IL-4 in serum at 2 and 3 weeks PV. Both levels of IFN-γ and IL-4 were significantly increased in the chickens received rHA-CVCVA5 or Re6-CVCVA5 vaccine, and slightly increased in the rHA vaccine group (Figures 5A,B). The increased magnitude of IFN-γ was higher than that of the IL-4.

Cytotoxic T lymphocytes play an important role in the control of virus infections. The adjuvanted rHA and Re6 vaccines elicited a robust CTL response compared to those of the non-adjuvanted rHA and Re6 vaccines. Further analysis demonstrated that vaccination of chickens with the adjuvanted rHA vaccine induced slightly higher levels of the CTL responses than those by vaccination with Re6 vaccines in the presence or absence of adjuvants. The CTL immune responses from the Re6 vaccine groups were low which was similar to that of the control groups (Figures 5C,D).

### H5 rHA VLPs Vaccine Confers Cross-Protection against Heterogeneous Strains

**Figure 6:**
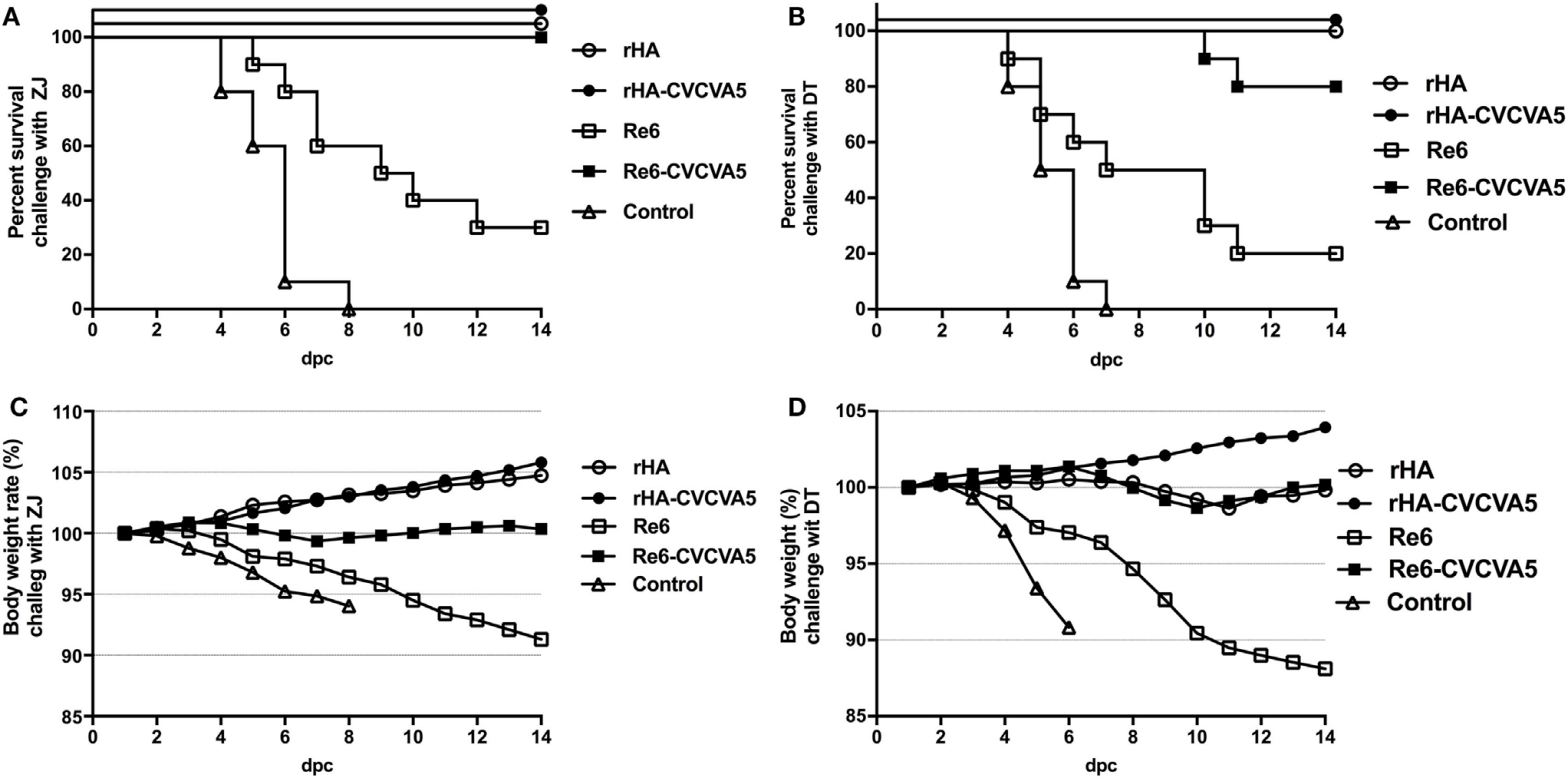
Survival rates and body weight changes of the specific pathogen-free (SPF) chickens after heterologous virus challenge. At 3 weeks postvaccination, groups of SPF chickens (*n* = 10) were intranasally challenged with 100 LD_50_ viruses of ZJ (A/Chicken/Zhejiang/2011, H5N2, subclade of 2.3.4.6) **(A)**, or DT (A/Chicken/Huadong/4/2008, H5N, clade 7) **(B)**, and the clinical symptoms or death, and body weight changes were daily monitored and recorded for 14 days post challenge **(C,D)**.

To investigate whether rHA vaccine could confer cross-protection against a lethal challenge with heterogeneous virus, the rHA-vaccinated chickens were challenged with ZJ (clade 2.3.4.6) and DT (clade 7) viruses, respectively (Figure 6). After a lethal ZJ virus challenge, all birds survived in vaccine groups of rHA, rHA-CVCVA5, and Re6-CVCVA5 (Figure 6A). In contrast, only 30% (3/10) chickens survived in the Re6 vaccine group, and all chickens died of infection in the control group. In the challenge test with DT virus, all chickens survived the infection in the rHA and the rHA plus CVCVA5 vaccine groups. Two out of ten chickens were dead in the groups of Re6-CVCVA5. Only two out of ten chickens survived in the Re6 (clade 2.3.2) vaccine group and no chicken survived in the control group (Figure 6B).

After challenge with ZJ virus, the body weight still slightly increased in chickens received with the rHA and the rHA-CVCVA5 vaccines. The birds in the rHA-CVCVA5 vaccine group gained the most weight among all challenge groups. The body weights of the chickens were nearly unchanged in the Re6-CVCVA5 vaccine group during 14 days of the monitoring period. In contrast, the chickens significantly lost weight in the Re6 vaccine and the control groups after challenge with ZJ virus (Figure 6C). However, only the rHA-CVCVA5 vaccine group showed a weight gain after challenge with DT virus. The birds in the rHA VLPs and Re6-CVCVA5 groups slightly lost weight at day 8–11, and then quickly recovered to initial weight during the 14-day of observation period. Similar to the challenge with ZJ virus, the chickens also exhibited rapid weight loss in the groups of the Re6 vaccine and control after challenge with DT virus (Figure 6D).

The excreted viruses *via* oropharynx and cloaca were analyzed to determine the virus replication at 3, 5, and 7 days PC with ZJ and DT viruses (Table 1). After ZJ virus challenge, virus shedding was not detected in chickens from the rHA-CVCVA5 vaccine group, and one bird was positive of virus isolation in both the rHA and the Re6-CVCVA5 groups. All birds were shedding viruses in the Re6 vaccine and control groups. A similar pattern of virus shedding was observed after DT virus challenge (Table 1). Chickens in the rHA-CVCVA5 group were free from virus detection. Three chickens in the rHA group and four chickens in the Re6-CVCVA5 group recovered viruses from swab samples. All birds in the Re6 vaccine and control groups excreted viruses PC.

**Table 1:** Virus shedding and clinical symptoms after challenge of chickens.

Group	Challenge virus	Oropharyngeal swab (virus shedding number/total number)			Cloacal swab (virus shedding number/total number)			Total virus shedding number/total number	No. clinical symptoms

		3 dpc	5 dpc	7 dpc	3 dpc	5 dpc	7 dpc		
rHA	ZJ	0/10	1/10	1/10	0/10	1/10	1/10	1/10	0
rHA-CVCVA5	ZJ	0/10	0/10	0/10	0/10	0/10	0/10	0/10	0
Re6	ZJ	3/10	3/10	6/10	3/10	6/10	10/10	10/10	9
Re6-CVCVA5	ZJ	0/10	1/10	0/10	0/10	1/10	1/10	1/10	0
Control	ZJ	10/10	10/10	10/10	10/10	10/10	10/10	10/10	10
rHA	DT	2/10	2/10	3/10	2/10	3/10	3/10	3/10	0
rHA-CVCVA5	DT	0/10	0/10	0/10	0/10	0/10	0/10	0/10	0
Re6	DT	5/10	6/10	7/10	4/10	7/10	10/10	10/10	9
Re6-CVCVA5	DT	1/10	2/10	3/10	2/10	3/10	4/10	4/10	4
Control	DT	10/10	10/10	10/10	10/10	10/10	10/10	10/10	10

*ZJ virus is the abbreviation virus strain name of A/Chicken/Zhejiang/2011 (H5N2), attributed to the subclade of 2.3.4.6, and DT is virus of A/Chicken/Huadong/4/2008 (H5N1), clade 7. The oropharyngeal and cloacal swab samples were collected at 3, 5, and 7 days post challenge. Virus positivity or shedding was determined by inoculating each swab solution into 3 eggs of 10-day-old specific pathogen-free chicken embryos. The virus isolation positive swab means one or more of the inoculated egg allantoic fluids with reciprocal of hemagglutination titers higher than 4. rHA, H5 rHA VLPs vaccine. Re6, a commercial vaccine strain with HA and neuraminidase from A/duck/Guangdong/S1322/2006 (H5N1, clade 2.3.2) and six inner genes from PR8 (H1N1)*.

## Discussion

In this study, we investigated immune responses induced by consensus HA sequence-based VLPs vaccine (rHA) in chickens, including serological and mucosal HI antibodies, cytokines, and induction of cross-protection against two heterologous subclades of H5 subtype AI virus challenge.

Development of vaccine based on the consensus HA sequences is one of the potential strategies to induce broadly protective immune responses against influenza viruses with high mutation rates and antigenic diversities. Consensus sequences encode the most common residues found at each position for the selected population, which are compatible with the site mutation residues in different clade viruses. We have compared the consensus HA sequence from the amino acid residues with that from the nucleotide level, and there were slightly differences mainly located at 310–340 amino acid residues (data not shown). It is expected that consensus sequences at nucleotide levels would be stable in mRNA translation. Besides, we have developed a wild-type Re6 HA-based vaccine which was poorly immunogenic and conferred partial protection against challenge with ZJ and DT viruses (data not published). In contrast to the single virus strain sequence-based subunit vaccine providing strain-restricted immunity, the consensus-based vaccines have been previously investigated as a strategy for eliciting broadly reactive immune responses for different pathogens, including chikungunya virus (25), hepatitis B virus (26), hepatitis C virus (27), Middle East respiratory virus (28), HIV-1 (29), and influenza (10, 30, 31).

The consensus rHA VLPs based vaccines elicited protective immune responses similar to those of the inactivated whole-virus vaccine, and could be applied to the assessment criteria of commercial inactivated virus vaccine, the index including serum HI titers and protective efficacy PC. We have also tested the efficacy of a single copy of the M2 ectodomain (M2e) as a broad-spectrum influenza vaccine candidate in chickens (22). Nevertheless, its further application was restricted by the lack of an appropriate method to evaluate the immune response elicited by the M2e antigens in chickens.

The rHA vaccine in the absence of CVCVA5 elicited higher levels of serological and mucosal antibody responses than those of the Re6 vaccine without CVCVA5 in terms of the HI antibody titers against the Re7 and Re8 antigens in both of the SPF and commercial chickens, and of the SN titers in DF-1 cells. However, higher levels of HI titers after Re6 vaccination were observed when using Re6 as a test antigen in both the SPF and commercial chickens because of an Re6 antigen exactly matching with the Re6 vaccine. In contrast to the HI titers, the neutralizing titers more closely reflect the capability of antibodies inhibiting the replication and propagation of virus. The results of humoral immune responses indicated that the rHA-based vaccine can elicit broad-spectrum antibodies reactive to different antigens, which is consistent with other influenza vaccines based on consensus HA sequences (10).

The VLPs vaccines were superior to the inactivated whole-virus vaccine in eliciting cell-mediated immune responses in animal models as reported in previous studies (17, 32, 33). The residual recombinant baculoviruses in the rHA vaccine preparations can activate Toll-like receptors, which correlated with cellular immune response. In this study, we have also monitored slightly higher levels of IFN-γ and CTL responses induced by the rHA VLPs vaccine in the absence of CVCVA5 compared to those of the Re6 vaccine without CVCVA5.

The challenge studies were carried out with two heterogeneous H5 subtype virus strains (ZJ and DT). The results from this study provide evidence that the rHA VLPs vaccines could be applicable to the field where there exist different subclades of H5 subtype viruses. In contrast to the Re6 vaccine in the absence of immunopotentiator, the rHA vaccine without immunopotentiator broadly protected the vaccinated chickens free from weight loss and death after infection with heterogeneous viruses. The multi-immune components including humoral antibodies, cytokine responses, and CTL activities elicited by the rHA vaccines with CVCVA5 adjuvants could contribute to broad protection against H5 subtype variants infection.

Besides construction of the broad-spectrum antigen, the adjuvant is another option to improve the efficacy of vaccines. The adjuvant, CVCVA5, was shown to be effective on improving the efficacy of the commercial inactivated monovalent or polyvalent vaccines in our previous studies (18, 19). Compared to the rHA and Re6 vaccines in the absence of adjuvant, CVCVA5 adjuvanted vaccine formulations moderately increased the serum antibody levels, significantly improved antibody levels in BAL, and also significantly enhanced the levels of CTL immune responses and cytokines such as IFN-γ and IL-4 in both adjuvanted rHA and Re6 vaccines. There is no significant difference in the protection rates among the groups of rHA vaccine with or without immunopotentiator, suggesting major roles of humoral antibodies in conferring cross-protection by consensus rHA vaccination. In contrast to the rHA vaccine, the increased cellular immune responses in the commercial Re6-CVCVA5 vaccine appear to play pivotal roles in providing effective protection against heterologous virus challenge when antibodies alone are insufficient for effective protection.

In this study, we have reported HA consensus sequence-based H5 rHA VLPs vaccines, which elicited a broad-spectrum serum and mucosal antibody response, and protected chickens experimentally challenged with different subclades of H5 subtype viruses. The results from this study support that development of subunit VLP vaccines based on consensus HA sequences conferring a broad-spectrum protection can provide a potential application in poultry farm with continuing antigenic drift variants.

## Ethics Statement

All animal studies were carried out in accordance with the recommendations in the National Guide for the Care and Use of Laboratory Animals. The protocol (VMRI-AP150306) was approved by the Review Board of National Research Center of Engineering and Technology for Veterinary Biologicals, Jiangsu Academy of Agricultural Sciences and Yangzhou University. The surgery and euthanasia were performed under anesthesia with sodium pentobarbital solution (100 mg/kg body weight) *via* intravenous route to minimize suffering. All experiments involved in the live H5 subtype viruses were performed in the biosafety level 3 laboratory facilities. At the end of experiments, the discarded live viruses, wastes, and infected animal carcasses were autoclaved and incinerated to eliminate biohazards.

## Author Contributions

Conceived and designed the experiments: YT, PW, JL, XZ, and XL. Performed the experiments: YT, PW, JL, XZ, MM, and LF. Analyzed the data: YT, PW, JL, XZ, XL, and DP. Contributed reagents/materials/analysis tools: YT, PW, JL, XZ, MM, LF, JH, and DP. Wrote and edited the paper: YT, PW, S-MK, DP, and XL. All authors read and approved the final manuscript.

## Conflict of Interest Statement

The authors declare that the research was conducted in the absence of any commercial or financial relationships that could be construed as a potential conflict of interest. YT, PW, JL, XZ, MM, LF, and JH are authors on a pending China patent application describing the culture and produce of rHA in bioreactor system (Title: Preparation method of the H5 subtype HA-based subunit vaccine. Reference number: 201710140542.2).
